# Supplementary material for: Resveratrol Contrasts IL-6 Pro-Growth Effects and Promotes Autophagy-Mediated Cancer Cell Dormancy in 3D Ovarian Cancer: Role of miR-1305 and of Its Target ARH-I
Source: Cancers (Basel). 2022 Apr 25;14(9):2142. doi: 10.3390/cancers14092142 (PMC9101105; doi:10.3390/cancers14092142)
Supplement: Supplementary file 1 [file cancers-14-02142-s001.zip › cancers-1691485-supplementary.pdf]

Figure 2 OVCAR3

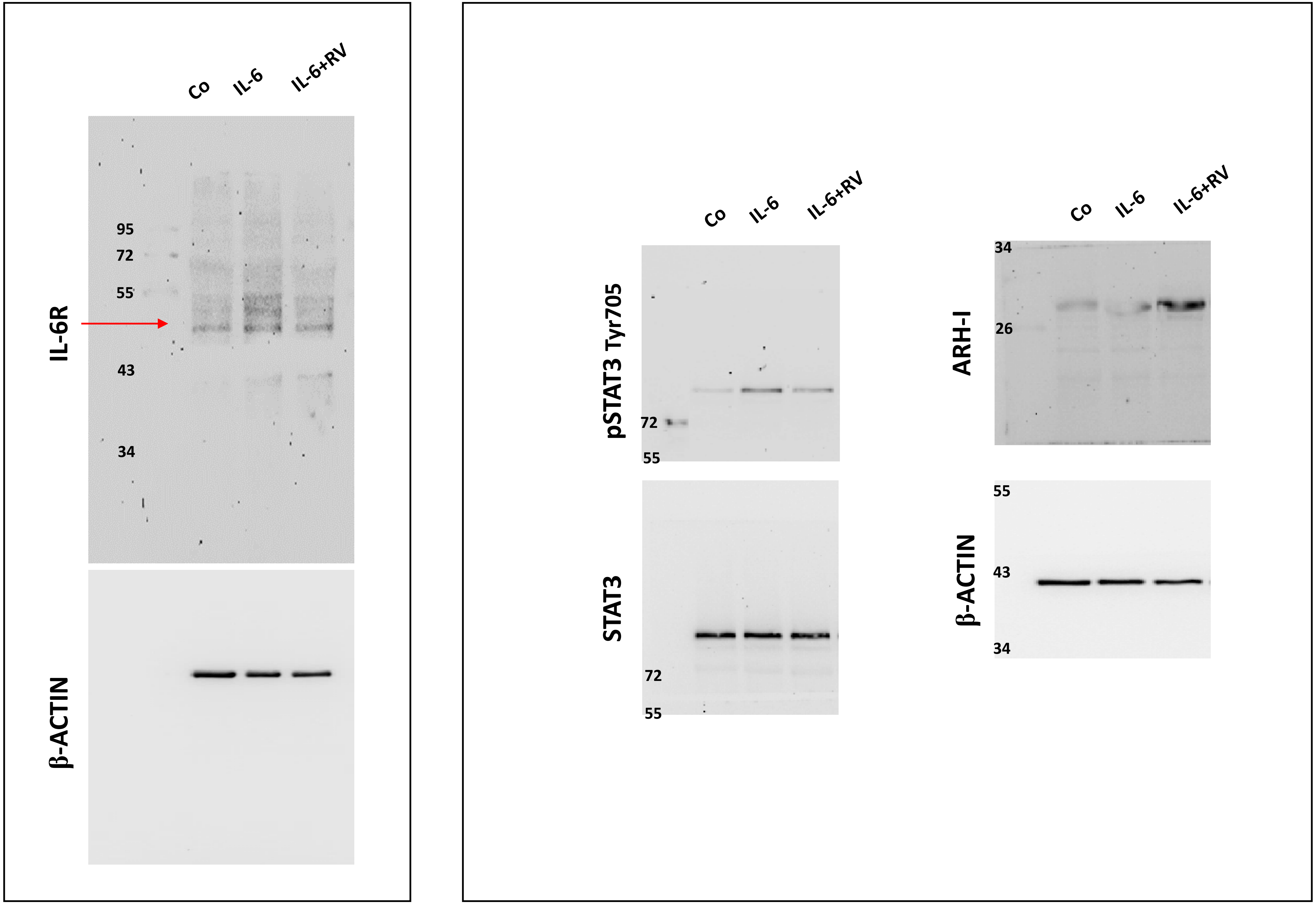

Figure 2 OAW42

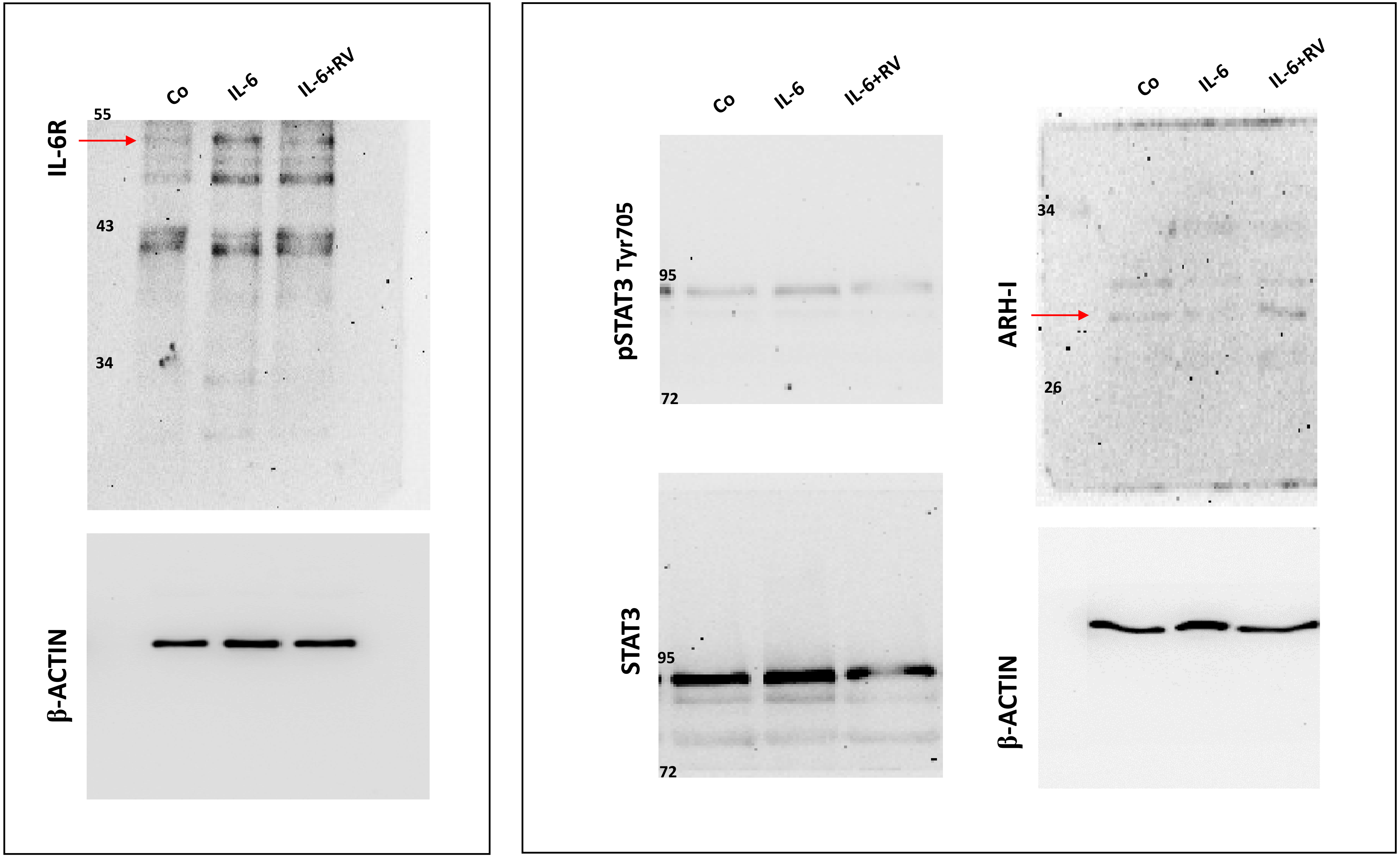

Figure 2 KURAMOCHI

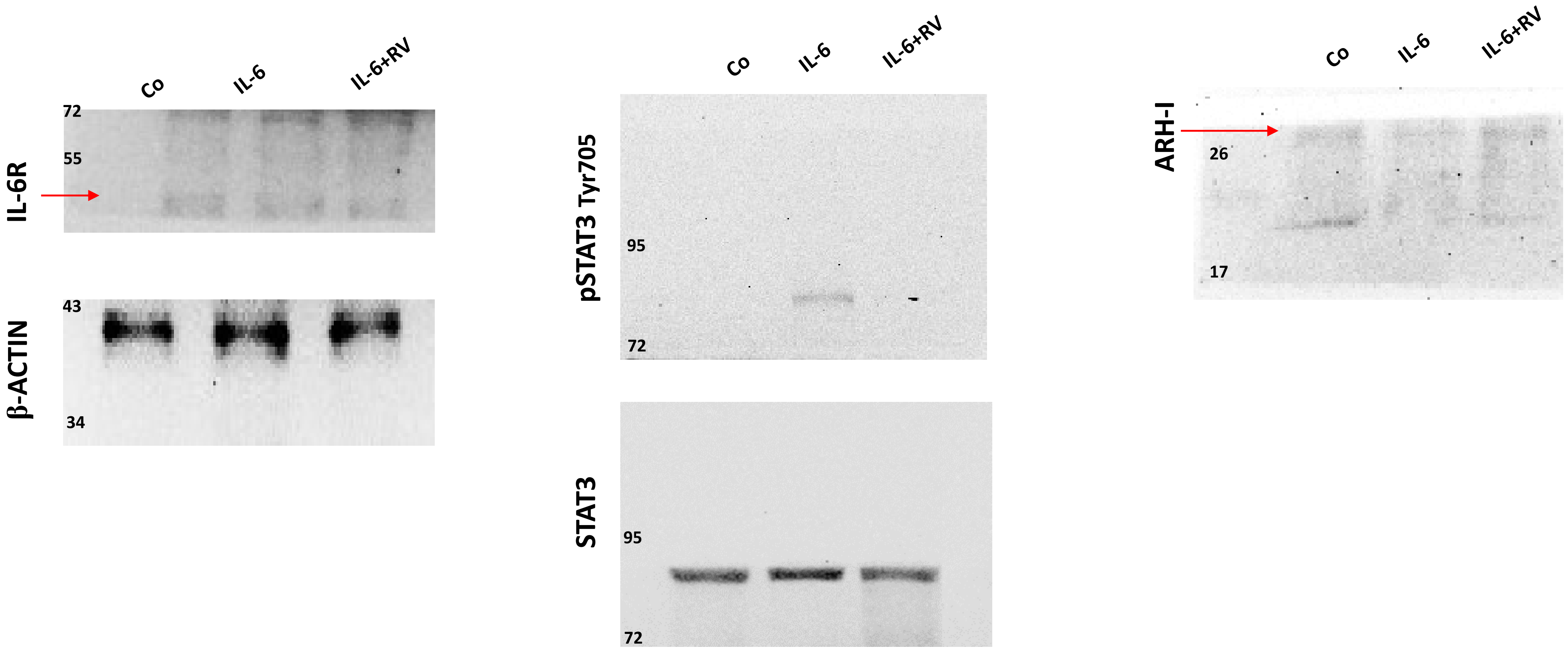

Figure 3C

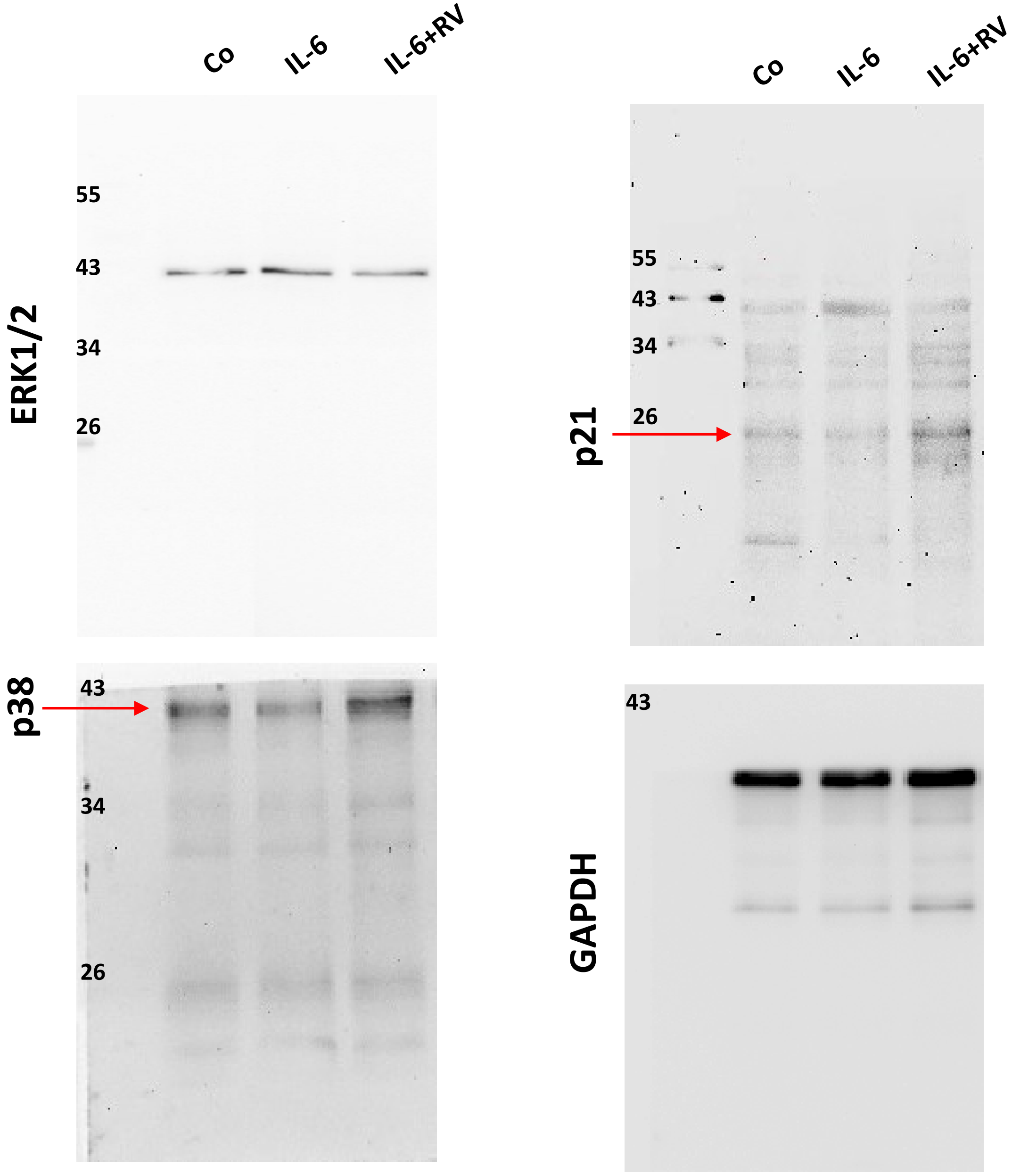
Co
IL-6
IL-6+RV
ERK1/2
55
43
34
26
p21
55
43
34
26
p38
43
34
26
GAPDH
43

Figure 5

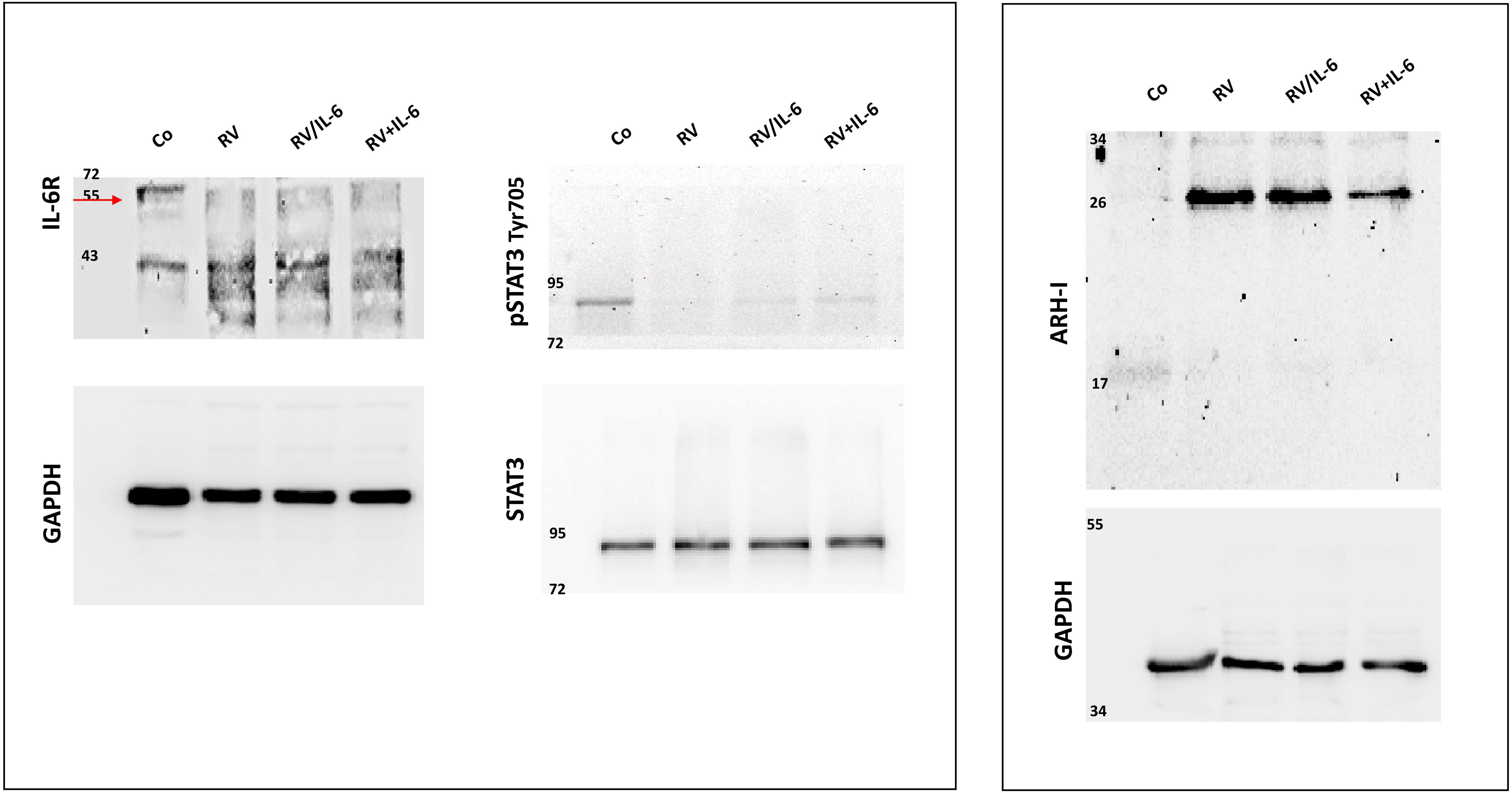
Co
RV
RV/IL-6
RV+IL-6
IL-6R
72
55
43
GAPDH
pSTAT3 Tyr705
95
72
STAT3
95
72
ARH-I
34
26
17
GAPDH
55
34

Figure 6C

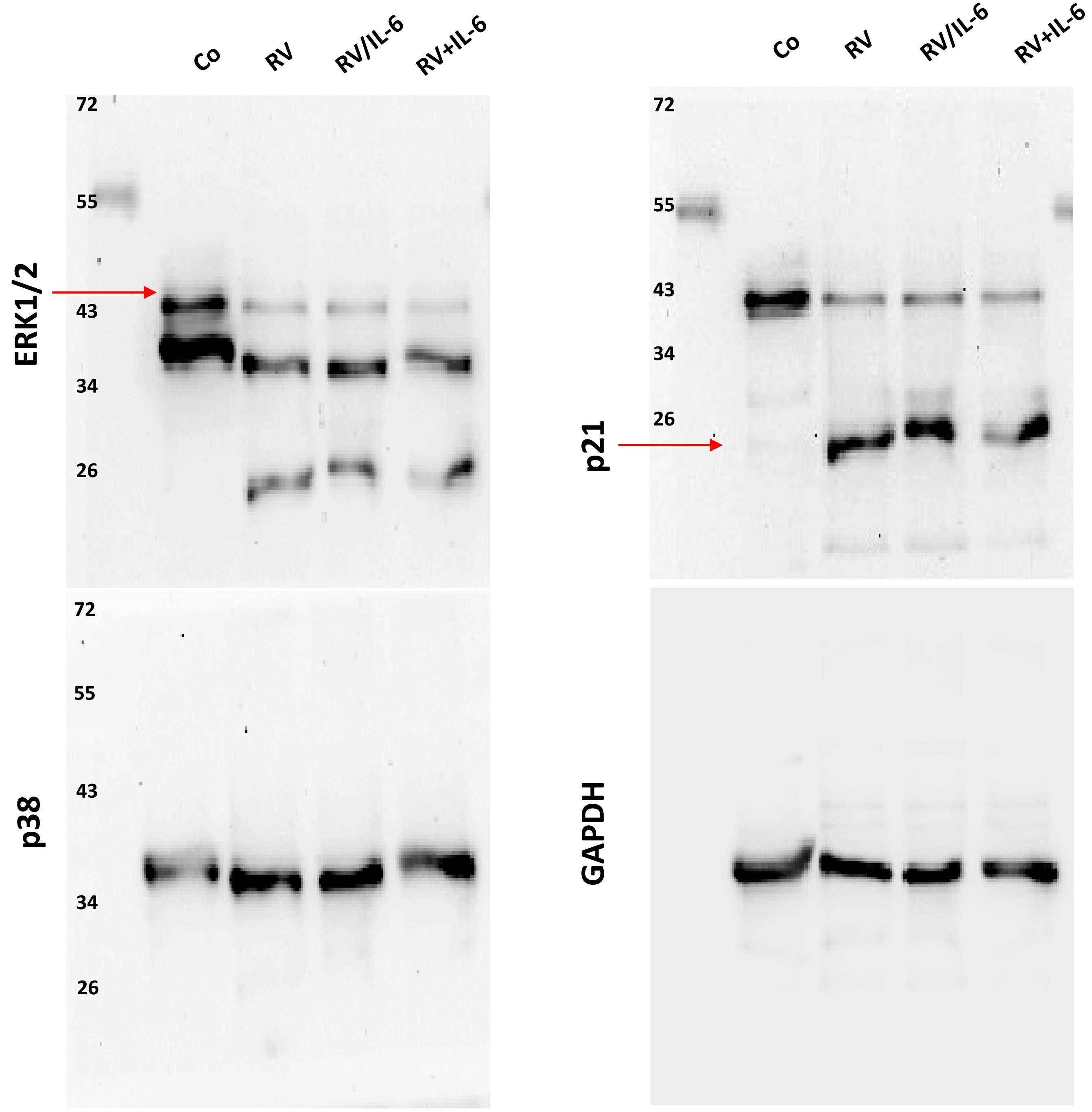

Figure 7A

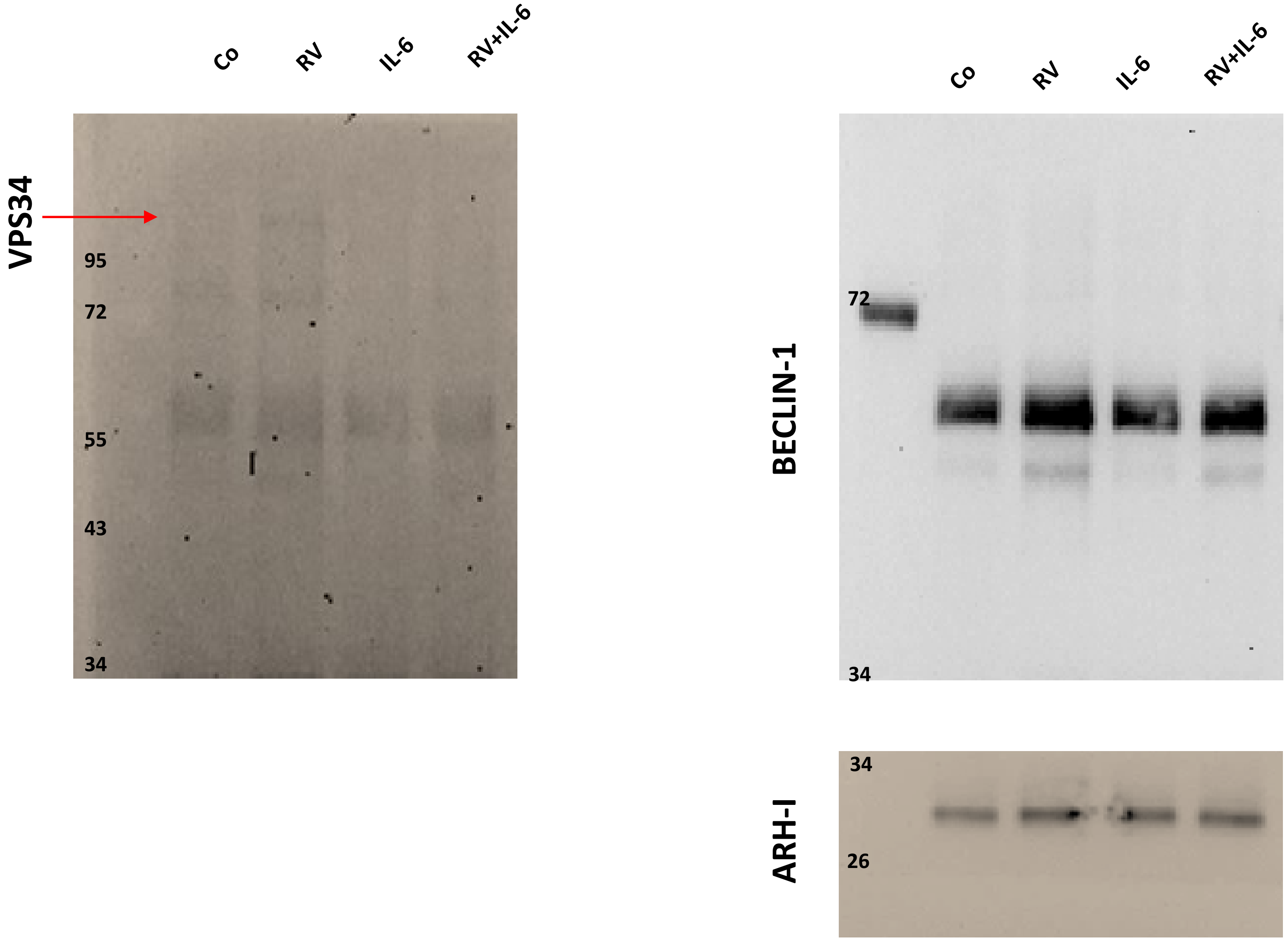

Co
RV
IL-6
RV+IL-6
VPS34
95
72
55
43
34
Co
RV
IL-6
RV+IL-6
BECLIN-1
72
34
ARH-I
34
26

Figure 7C

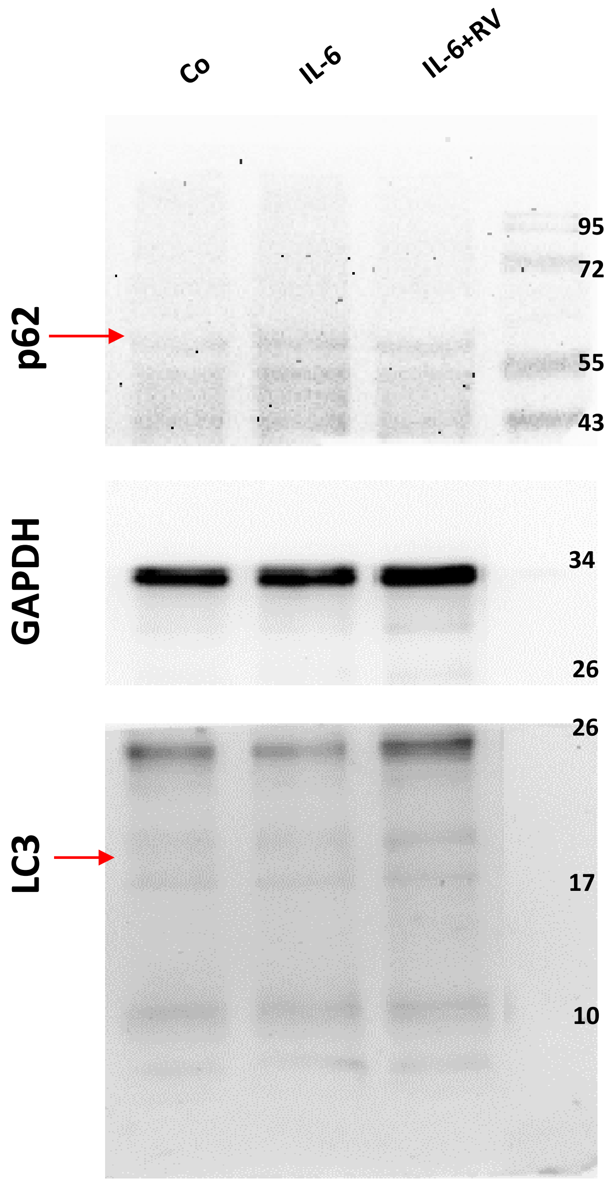

Figure 7E

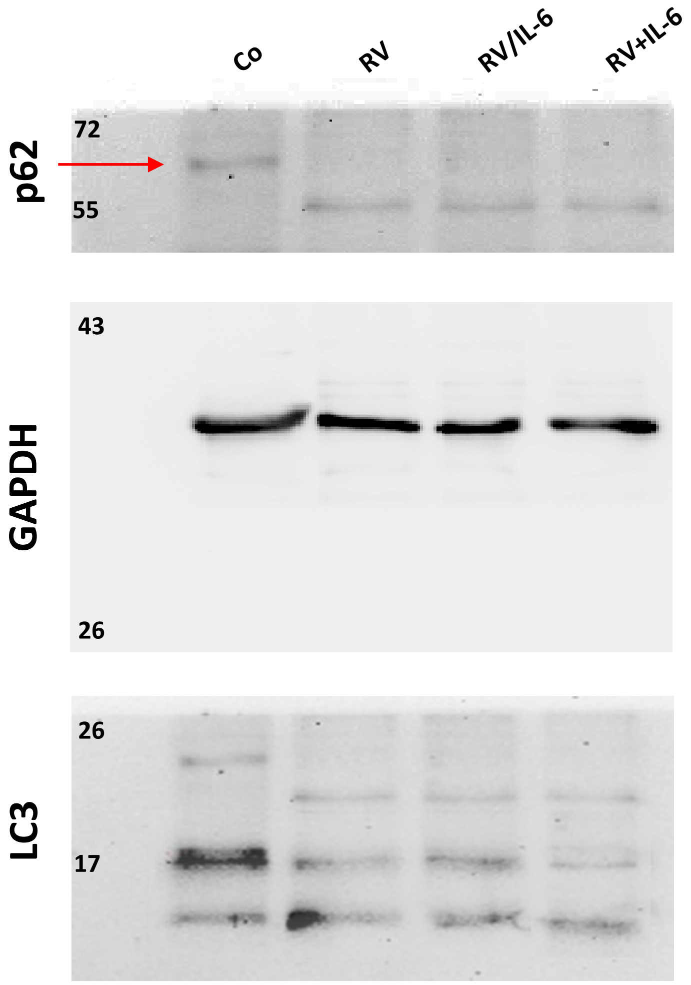

Figure 8F

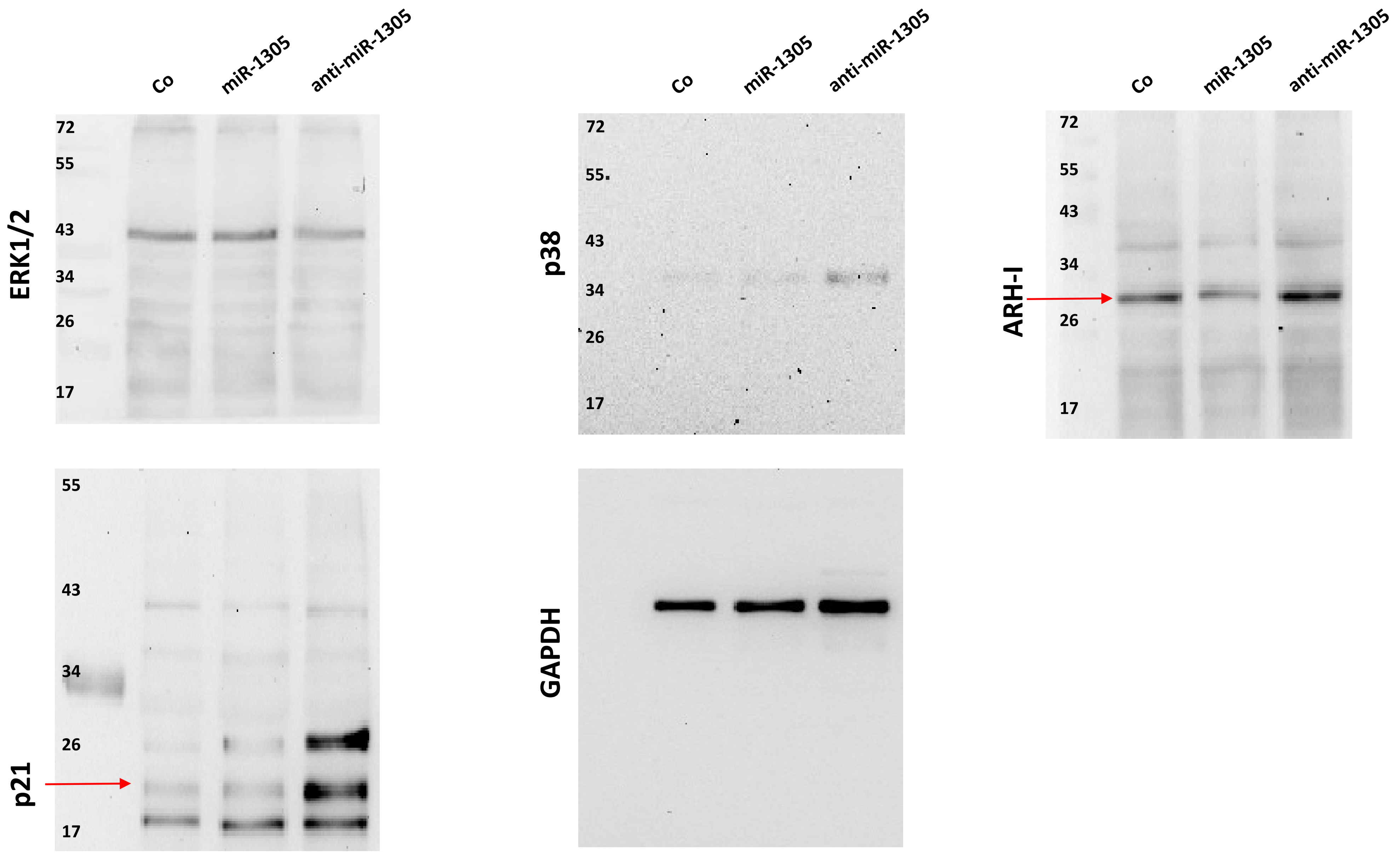
Co
miR-1305
anti-miR-1305
ERK1/2
72
55
43
34
26
17
p38
ARH-I
p21
GAPDH

Figure 9A

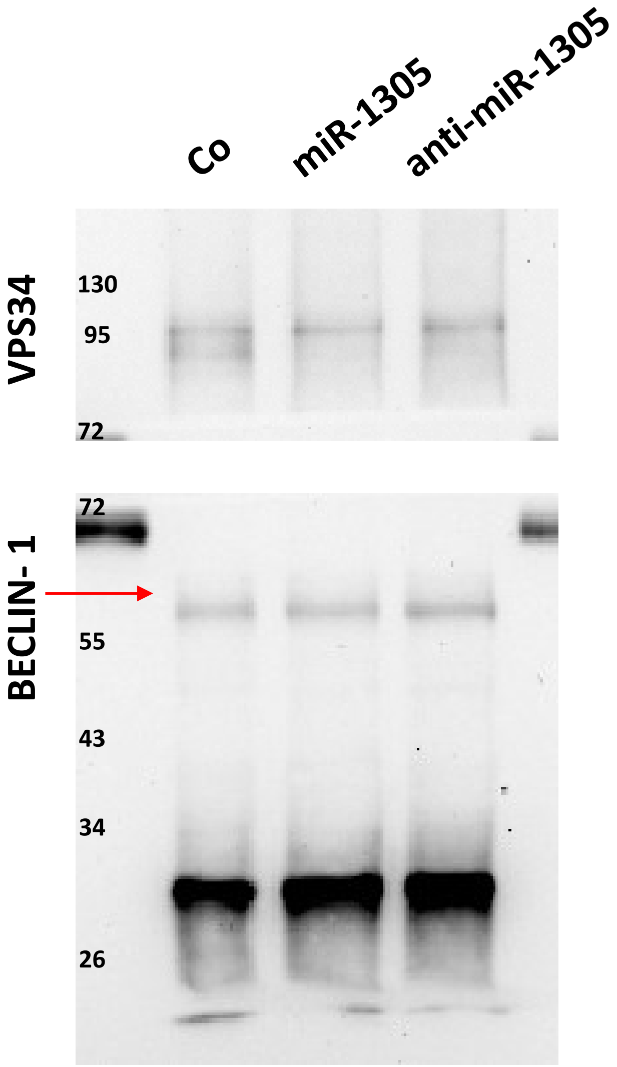

Co
miR-1305
anti-miR-1305
VPS34
130
95
72
72
BECLIN- 1
55
43
34
26

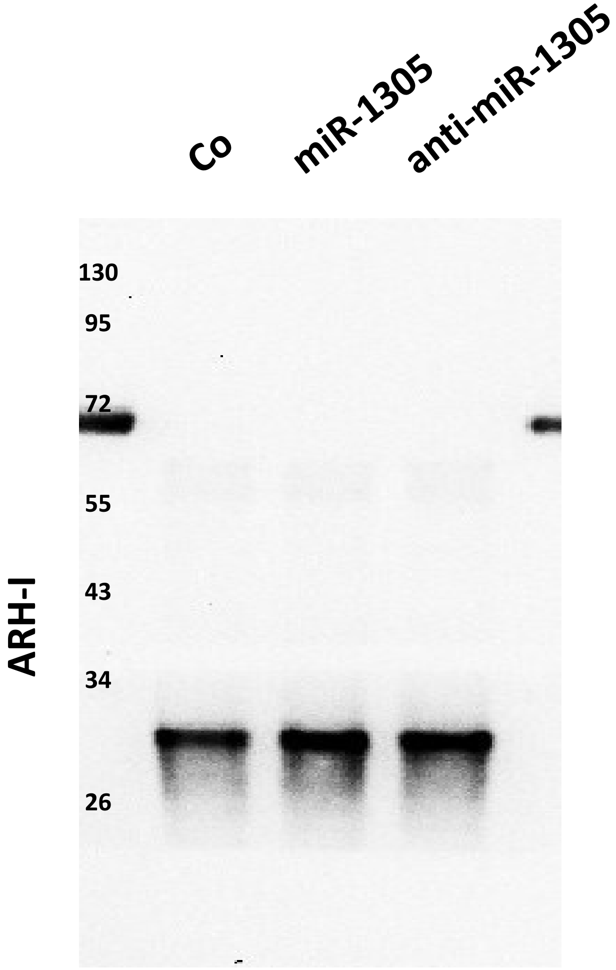

Co
miR-1305
anti-miR-1305
130
95
72
55
ARH-I
43
34
26

Figure 9B

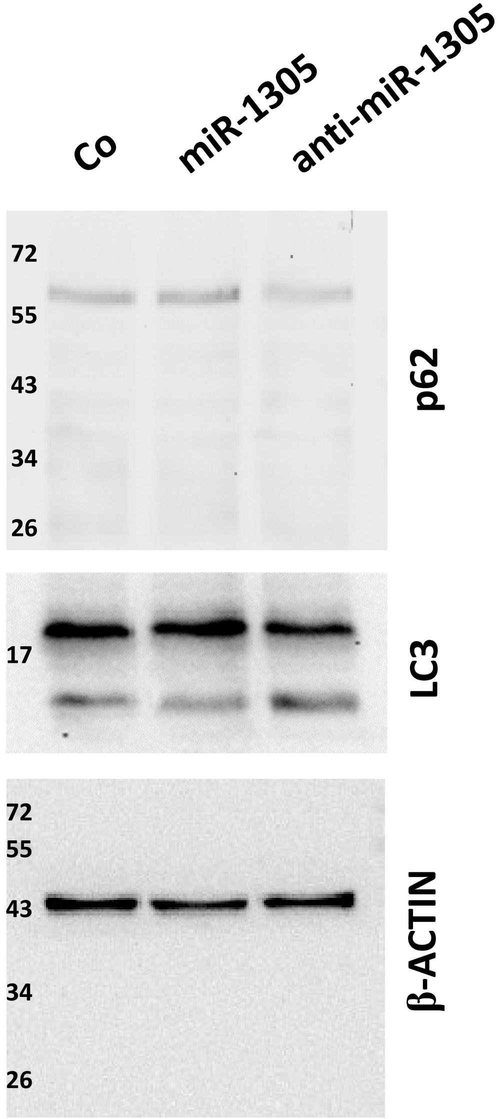
Co
miR-1305
anti-miR-1305
72
55
43
34
26
p62
17
LC3
72
55
43
34
26
β-ACTIN

Figure 9C

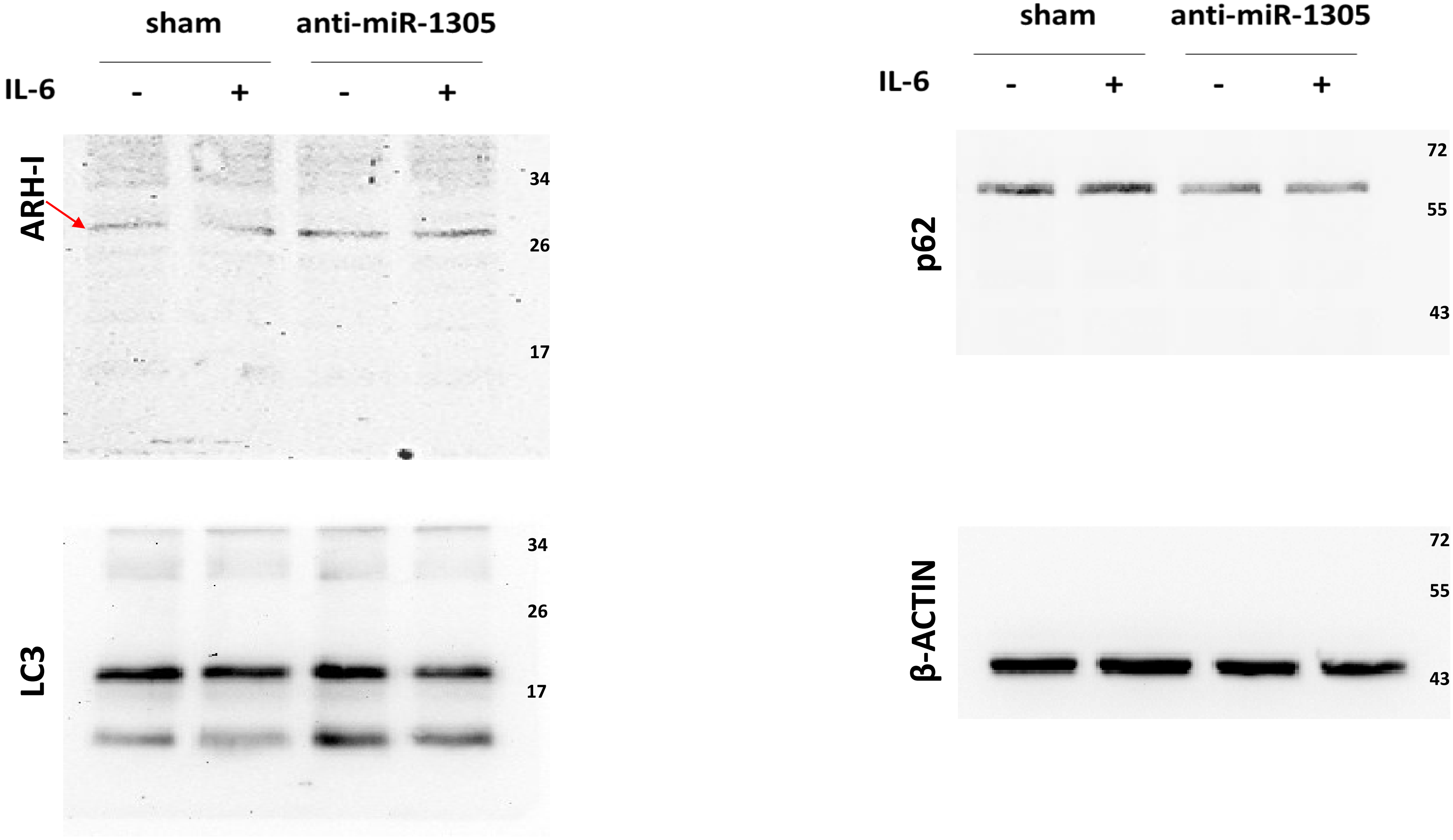

sham
anti-miR-1305
IL-6
-
+
-
+
ARH-I
34
26
17
LC3
34
26
17
sham
anti-miR-1305
IL-6
-
+
-
+
p62
72
55
43
β-ACTIN
72
55
43
